# What happens to old insecticide-treated nets after households use in Burkina Faso?

**DOI:** 10.1186/s12936-024-05181-w

**Published:** 2024-11-20

**Authors:** Aristide S. Hien, Hervé Hien, Fidèle Y. Bacyè, Herman Badolo, Alfred Tiono, Cheick O. Diallo, Serge M.A. Somda, Herman Bazié, Matilibou Guira, Nicolas Meda

**Affiliations:** 1 Institut de Recherches en Sciences de la Santé, Centre national de la recherche scientifique et technologique, Ouagadougou, Burkina Faso; 2 Centre Universitaire de Tenkodogo, Tenkodogo, Burkina Faso; 3 Institut National de Santé Publique, Ouagadougou, Burkina Faso; 4 Centre National de Formation et de Recherche sur le Paludisme, Ouagadougou, Burkina Faso; 5 Université Nazi Boni, Bobo-Dioulasso, Burkina Faso; 6 Programme National de Lutte contre le Paludisme, Ministère de la santé et de l’hygiène publique, Ouagadougou, Burkina Faso; 7 Unité de Formation et de Recherches en Sciences de la Santé, Université Joseph Ki-Zerbo, Ouagadougou, Burkina Faso

**Keywords:** Insecticide-treated nets, Disposal methods, Alternative uses, Climate zones, Burkina Faso

## Abstract

**Background:**

Insecticide-treated nets (ITNs) are the most commonly deployed tools for controlling malaria transmission in sub-Saharan Africa. However, some reports associate multiple alternative uses of nets with poor disposal practices, prompting this study to assess existing alternative uses and disposal practices of old ITNs in Burkina Faso after four universal distribution campaigns.

**Methods:**

A quantitative survey combined with qualitative data collection was used to describe existing alternative uses and disposal practices for old ITNs in households from selected study sites in the three climatic zones of Burkina Faso. A survey questionnaire was distributed to 3,780 participants, and 12 focus groups were held to elucidate responses regarding existing disposal practices and alternative uses of ITNs.

**Results:**

Of the 3780 households surveyed, 87.4% (3,330) reported having disposed of their ITNs when they were no longer usable due to age or wear. The most commonly cited disposal methods included alternative uses (67.4%), disposal with other garbage (61.4%), and burying (9%). In addition, the most common alternative uses included fencing for crops and seedlings (51.8%); ropes for tying items (40.4%); animal protection fencing (17.8%); house fencing (16.8%); bed covers (13.3%) and curtains for doors or windows (12.6%). Furthermore, trends in ITNs disposal mechanisms and alternative uses differed between study sites in each climate zone. All these ITNs disposal mechanisms and the different types of alternative use of old ITNs were confirmed in the focus group discussions.

**Conclusion:**

The findings underscore the need for comprehensive strategies to manage the disposal and repurposing of old ITNs in Burkina Faso. Addressing gaps in disposal guidelines, promoting safe and beneficial reuse practices, and actively involving communities in the decision-making process can help mitigate health risks associated with the improper disposal and repurposing of old insecticide-treated nets and improve the overall effectiveness of malaria control programmes. Through these efforts, both public health and environmental concerns can be addressed in a sustainable and collaborative manner.

## Background

In 2022, approximately 282 million insecticide-treated nets (ITNs) were distributed to countries affected by malaria, with around 92% going to sub-Saharan Africa [1]. The primary goal of using insecticide-treated nets (ITNs) is to protect people from mosquito bites, thereby reducing malaria and other mosquito-borne diseases [2]. The widespread distribution of ITNs could result in a rise in solid waste and environmental pollution in these countries, where waste management is already a major issue [3–5]. It is uncertain whether ITNs contribute to plastic waste or what their ultimate disposal and environmental impact might be. As the use of ITNs increases, it becomes increasingly important to identify the most effective methods for disposing of old nets once they are no longer needed. (repetition)

In many parts of Africa, ITNs are repurposed for various uses, such as fencing for vegetable gardens and chicken coops, sifting grain, and making fishing nets or other construction materials. These alternative uses have the potential to cause environmental harm. The insecticides (pyrethroids and pyrroles) incorporated into ITNs are of low toxicity to mammals [6], but improper disposal of ITNs can pose risks, including adverse effects on aquatic ecosystems and non-target species. For example, the insecticides can exert additional selection pressure on mosquitoes larva in the aquatic habitats [7]. The issue of environmental pollution by plastics remains a significant global challenge, with a range of potential consequences for aquatic life, wildlife, climate change, human health and economic development [8–12]. The World Health Organization (WHO) has highlighted concerns about the potential environmental impact of accumulated ITNs and their packaging in 2014 [13].

Reusing ITNs can lead to contamination of crops, vegetables, soil, and groundwater through several mechanisms by direct contact with plants, leaching into soil, contaminated run-off and groundwater pollution leading to long-term contamination of water sources relied upon by local communities [13]. Furthermore, open-air burning of these materials can release dangerous, persistent toxins, posing significant environmental and health risks [14]. The WHO has recommended several methods for disposing of or handling used insecticide-treated nets (ITNs), including: (1) continuing to use ITNs with holes until new ones are available; (2) avoiding disposal of used ITNs in bodies of water; (3) collecting used ITNs for disposal by the National Malaria Control Programme (NMCP); (4) incinerating used ITNs; and (5) developing guidelines, policies, and regulations in collaboration with national environmental authorities [13, 15]. It is crucial to determine whether local health and environmental authorities are aware of these guidelines and have the necessary capacity to implement and monitor them.

The lack of transparent data distribution, usage, and environmental impact from international bodies is due to communication difficulties. This absence of readily available information on the topic is a significant obstacle. There is a need for clear, authoritative guidance on the proper disposal of ITNs, as emphasized by researchers and practitioners. Evidence indicates that old ITNs are often misused and improperly disposed of within communities, including being repurposed for uses other than their intended purpose. The aim of this study was to evaluate the alternative uses and disposal practices for ITNs among community members in each of Burkina Faso’s climate zones. Similar works on ITN disposal in sub-Saharan Africa in 2020 where many households repurpose old ITNs for non-health-related uses, such as fishing, gardening, or fencing [16], on the assessment of ITN utilization in Burkina Faso in 2019 that found significant regional variations in ITN usage and disposal practices, influenced by socio-economic factors and cultural beliefs [17]. Moreover, studies on environmental impact of ITN disposal highlighted significant challenges in the proper disposal of old long-lasting insecticidal nets and improper disposal practices pose environmental risks [18, 19]. However, the current literature on the disposal and alternative use of insecticide-treated nets (ITNs) presents several gaps. One of the major limitations is the narrow geographic scope of existing research, which often overlooks diverse climate zones and cultural contexts, particularly in West African countries like Burkina Faso. Furthermore, socio-demographic factors such as education level, income status, and residency (urban vs. rural) have not been adequately explored, limiting the understanding of how these variables affect disposal behaviours and practices. Addressing these gaps is essential for developing more effective and context-specific strategies.

Burkina Faso faces the challenge of unsanitary conditions due to waste from used mosquito nets, much like other sub-Saharan African countries. Since 2010, the NMCP has conducted four nationwide distributions of mosquito nets. However, there has been no follow-up after these distributions to understand what happens to the old ITNs at the end of their lifecycle and how the community manages them and what is the socio demographic factors influencing the disposal methods and improper alternative use of old ITN. Therefore, it would be beneficial for decision-makers and policymakers to understand the various disposal practices and alternative uses of old nets to enhance management strategies.

## Methods

### Study area

**Fig. 1:**
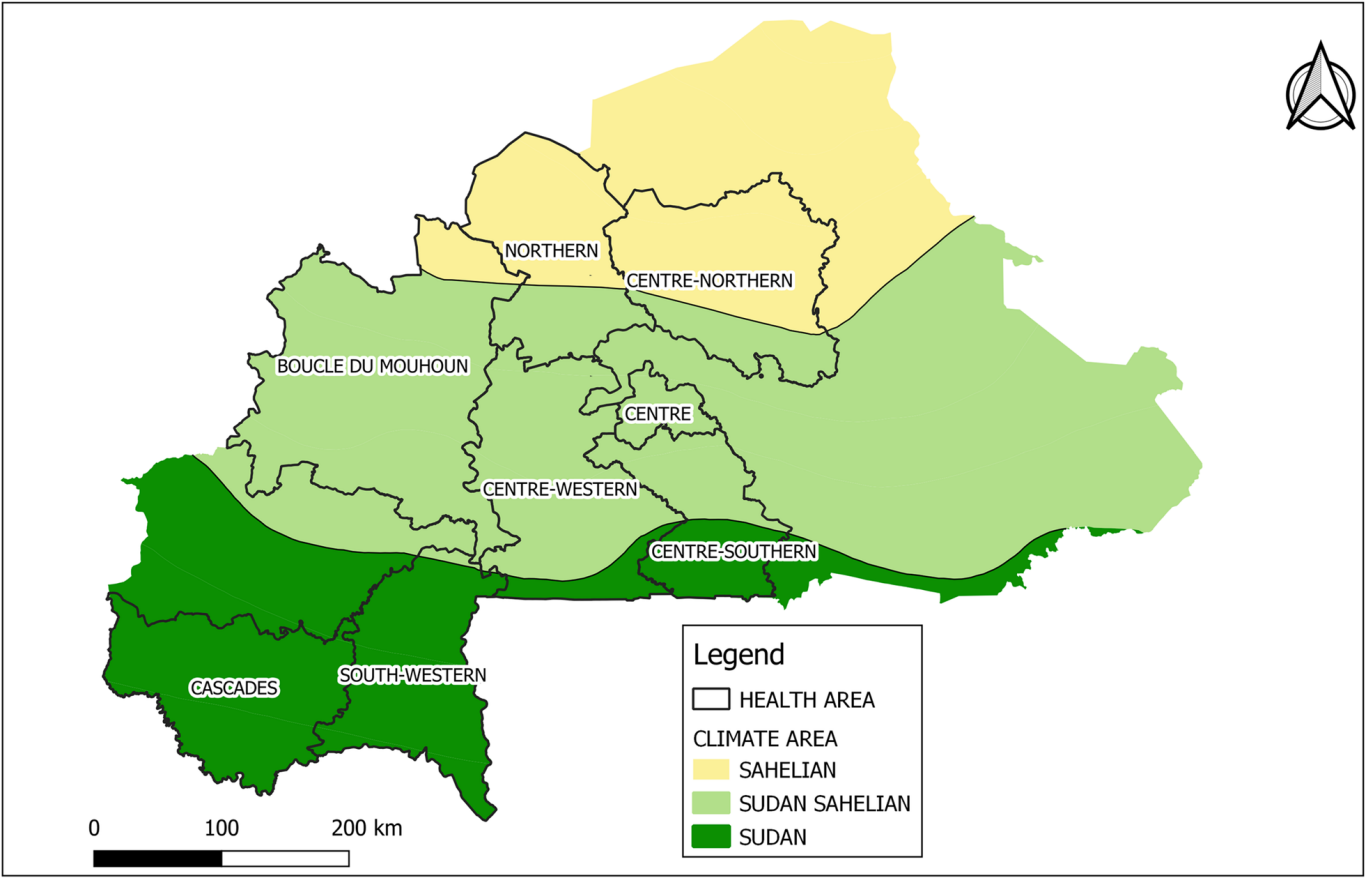
Study area, Burkina Faso

The study was conducted across eight health regions in Burkina Faso, covering the three-climate zone of the country: Sahelian (red), Sudan-Sahelian (orange), and Sudan (green) (Fig. 1). The Sudan climate zone, which includes the Cascades, Southwestern, and part of the Centre-Southern regions, is the wettest, with average annual rainfall between 1000 and 1200 mm, mostly occurring during the rainy season from May to November. The Sahelian zone, located in the Northern and Centre-Northern regions, is relatively dry, receiving less than 600 mm of rainfall annually. In the Sudan-Sahelian zone, which encompasses Boucle du Mouhoun, Centre-Western, Centre, and part of the Centre-Southern regions, the annual rainfall averages between 600 and 900 mm, with a shorter rainy season from June to September. Additionally, within each climate zone, residents share similar characteristics regarding climate, environment, and behaviour [11].

Recent data from the Burkina Faso Demographic and Health Surveys revealed that 86.6% of households nationwide are headed by men [20]. The average household size is 6.2 members, with urban areas having slightly smaller households (5.2 members) compared to rural areas (6.8 members). Malaria indicators show that, although the majority of insecticide-treated nets (ITNs) (82%) are provided through universal distribution campaign organized by the NMCP, 82.8% of households across the country have at least one ITN [20]. However, only 41% of households have at least one ITN for every two people who stayed in the household the previous night. Furthermore, 61.3% of the population reported sleeping under an ITN the previous night, including 67% of children under 5 and 71% of pregnant women [20].

### Study design

Cross-sectional surveys were conducted between February and March 2023, six months after the fourth universal campaign of nets distribution in Burkina Faso (2022–2025). Participants were recruited from each health region over a two-week period, from February 10 to 24, 2023, before the questionnaires were administered. Data collection focused on old ITNs distributed during the third campaign (2019–2022) within the community.

The study used a mixed-methods approach, where quantitative data analysis informed the themes of the qualitative study. It consisted of two components: (1) a quantitative survey was carried out to determine the required household size per climate zone, to which the questionnaires were administered, (2) Focus group discussions (FGDs) were conducted with community members selected from the survey respondents. These FGDs included equal numbers of male and female groups in each climate zone. The qualitative findings provided additional insights and clarification on the responses from the initial survey.

The combined results from both the qualitative and quantitative research were used to develop recommendations for managing old ITNs, which were then presented to the NMCP and donors.

### Sample size

The sample size for the survey questionnaire was determined using STATA software version 10.1. To calculate the required sample size for the quantitative data collection, a likelihood ratio test (LRT) was conducted. The first and second type errors were set at 5% and 20%, respectively, to balance precision with logistical feasibility for the survey. Additionally, the ratio between the two indicators was set at 1.09, resulting in the selection of f 1166 households per eco-climate area. To account for potential data loss, the sample size was increased to 1260 households. After adjusting the sample size, a total of 3,780 households were randomly selected for the study. For the qualitative data, the sampling included 12 focus group discussions (FGDs), six with male participants and six with female participants with each group consisting of 12 participants, totaling 144 participants. The study participants were selected based on the following criteria: (1) male or female heads of household or their representatives aged 18 years or older, and (2) individuals who had an insecticide-treated net (ITN) in use for alternative purposes within their compound or garden.

### Concept definitions

*Old insecticide-treated net* is defined as a net that has been used for a considerable period and may no longer be effective in providing protection against mosquitoes.

*Alternative use of old insecticide-treated net* is defined as the repurposing of mosquito nets that are no longer effective in protecting against mosquito bites but are still used for other functions or purposes.

The *disposal of old insecticide-treated nets* refers to the methods and practices used to manage insecticide-treated nets that are no longer useful for their intended purpose, such as preventing mosquito bites.

A *repurposed net* refers to an insecticide-treated net (ITN) that, after no longer being suitable for its primary purpose of mosquito bite protection, is used for alternative functions.

*Inactive net* is a net that is no longer effective at providing protection against mosquito bites due to damage, wear, or the degradation of insecticide. This type of net is typically no longer suitable for its intended purpose of mosquito control.

*Active net* refers to an insecticide-treated net (ITN) that remains effective for its primary purpose of providing protection against mosquito bites.

*Other uses* refer to any alternative uses of ITNs that do not fall into the primary or more frequently observed categories.

### Data collection procedures

The household questionnaire was administered to either the head of the household or another adult member (their representative) to gather information on the disposal of old ITNs and their alternative use within the household. Data on the disposal methods and alternative uses of old ITNs were collected through: (1) direct observation by the field team around the household; (2) self-reports from household interviews; and (3) community reporting on both aspects during focus group discussions (FGDs).

The questionnaires about quantitative data collection examined the existing disposal methods and alternative use of old ITN in country, including fishing, drying fish, covering/protecting seedlings/crops, curtains/screens for windows/doors, clothing, bed sheets/padding, sealing other nets, fencing, rope/ties, and protecting pets. In addition, respondents were asked to specify when the net was used for purposes other than sleeping, as well as for any other reasons. The questionnaires were translated in advance into the three main local languages of each community: Moore, Dioula, and Fulfulde.

Twelve focus groups were organized immediately after the quantitative data collection, once the main themes were identified. The objective was to clarify the findings from the quantitative component. The focus group discussion (FGD) guide was initially tested, and the questions were revised based on the results. It was then translated into the three major local languages of each climate zone. The discussions took place at local primary schools or public spaces within the community. Participants were carefully selected to ensure that the sample was representative of the broader population. Each climate zone had four focus groups: two for males and two for females, with 12 participants in each session. To maximize participation, males and females were separated during the discussions. These focus groups provided in-depth insights into community members’ understanding and perceptions of alternative uses and disposal practices of old ITNs. The discussions focused on participants’ experiences and views on ITN disposal and alternative uses, with input from each climate zone (Sudan, Sudan-Sahelian, and Sahelian). The discussions were audio-recorded and detailed notes were taken [19, 21, 22].

### Data analysis

All survey data were extracted using CSPro software (Version 7.7), then checked and cleaned in Excel, and finally coded in STATA statistical software (Version 10.1). The main study variables included: types of disposal methods for old ITNs as well as self-reported by respondents; the predominant old ITN disposal methods observed by the field team around households, categorized by climate zone; direct observations of type of alternative uses of old ITNs; and self-reported alternative uses of ITNs by respondents also categorized by climate zone. The disposal method of ITNs was recorded as a binary Yes/No outcome, while old ITNs were classified as either active or alternatively used. The findings from the quantitative survey were used to develop the focus group discussion (FGD) guides, which subsequently informed the deductive coding process. The findings were presented in accordance with the integration principles and practices typically employed in mixed-methods designs, as outlined by Madumla et al. [23]. The quantitative findings from the survey allowed to determine the most common disposal methods and alternative uses of old ITNs. These different uses were dependent variable in exploratory analysis. The main objective of exploratory analysis component was to assess the socio-demographic factors associated with (1) most common disposal methods and alternative uses of old insecticide-treated nets (ITNs). The analysis included both univariate and multivariate analyses to examine the relationships between independent variables (education status, residence, climate zone) and the dependent outcomes using Odds Ratios (OR) and Adjusted Odds Ratios (aOR) with 95% Confidence Intervals (CI). The reference group for each independent variable was “higher education” for education status, “urban” for residence and “Sudan zone” for climate zone. The univariate logistic regression was used to calculate crude Odds Ratios (OR) and 95% Confidence Intervals (CI) for each independent variable. Furthermore, to adjust for potential confounding factors and assess the independent effect of socio-demographic variables a multivariate logistic regression was conducted for each outcome (most common disposal methods and alternative use of old ITN) to calculate Adjusted Odds Ratios (AORs) and 95% Confidence Intervals (CI) adjusting for all independent variables (education, residence, climate zone). In multivariate analysis, the confounding factors were “age” and “wealth index”. In all analysis, p-values < 0.05 was considered statistically significant.

## Results

### Socio-demographic characteristics of study participants

A total of 3780 households were surveyed, evenly distributed across the three climate zones (1260 per zone) (Table 1). Of these, 2520 households (66.7%) were in rural areas. The average age of respondents was 32.5 years. Heads of households were predominantly male, with proportions varying by climate zone: 84.5% in the Sahelian zone, 80.3% in the Sudano-Sahelian zone, and 73.1% in the Sudanese zone. Additionally, 52.5% of respondents had no formal education (Table 1). Most households were classified as either very poor (37.1%) or poor (37.9%), while the wealthiest households constituted only 1% of the study population. These classifications are based on the wealth index, which measures household assets, housing characteristics, and access to services to rank households into wealth quintiles.

**Table 1:** Socio-demographic details of study participants

Variables	Frequencies (*n* = 3780)	Percentage (%)
Gender distribution		
Male	1606	42.5
Female	2174	57.5
***Age groups (years)***		
18–24	1043	27.6
25–34	1206	31.9
35–44	97	25.8
>=45	556	14.7
***Education status***		
No formal education	1984	52.5
Primary	1217	32.2
Secondary	466	10.9
Higher	113	2.9
***Wealth index***		
Very poor	1404	37.1
Poor	1431	37.9
Middle	687	18.2
Rich	222	5.9
Very rich	36	1
***Distribution across climate zones***		
Sudan	1260	33.3
Sudan-sahelian	1260	33.3
Sahelian	1260	33.3
***Place of residence according to territorial administration***		
Urban	1260	33.3
Rural	2520	66.7
***Head of household according to sex***		
Male	*N* = 2997	79.3
Sudan	907	30.3
Sudan-sahelian	1005	33.5
Sahelian	1085	36.2
Female	*N* = 783	20.7
Sudan	333	42.5
Sudan-sahelian	247	31.5
Sahelian	203	26

### Direct observation of and self-reported disposal methods for old ITNs by community

**Fig. 2:**
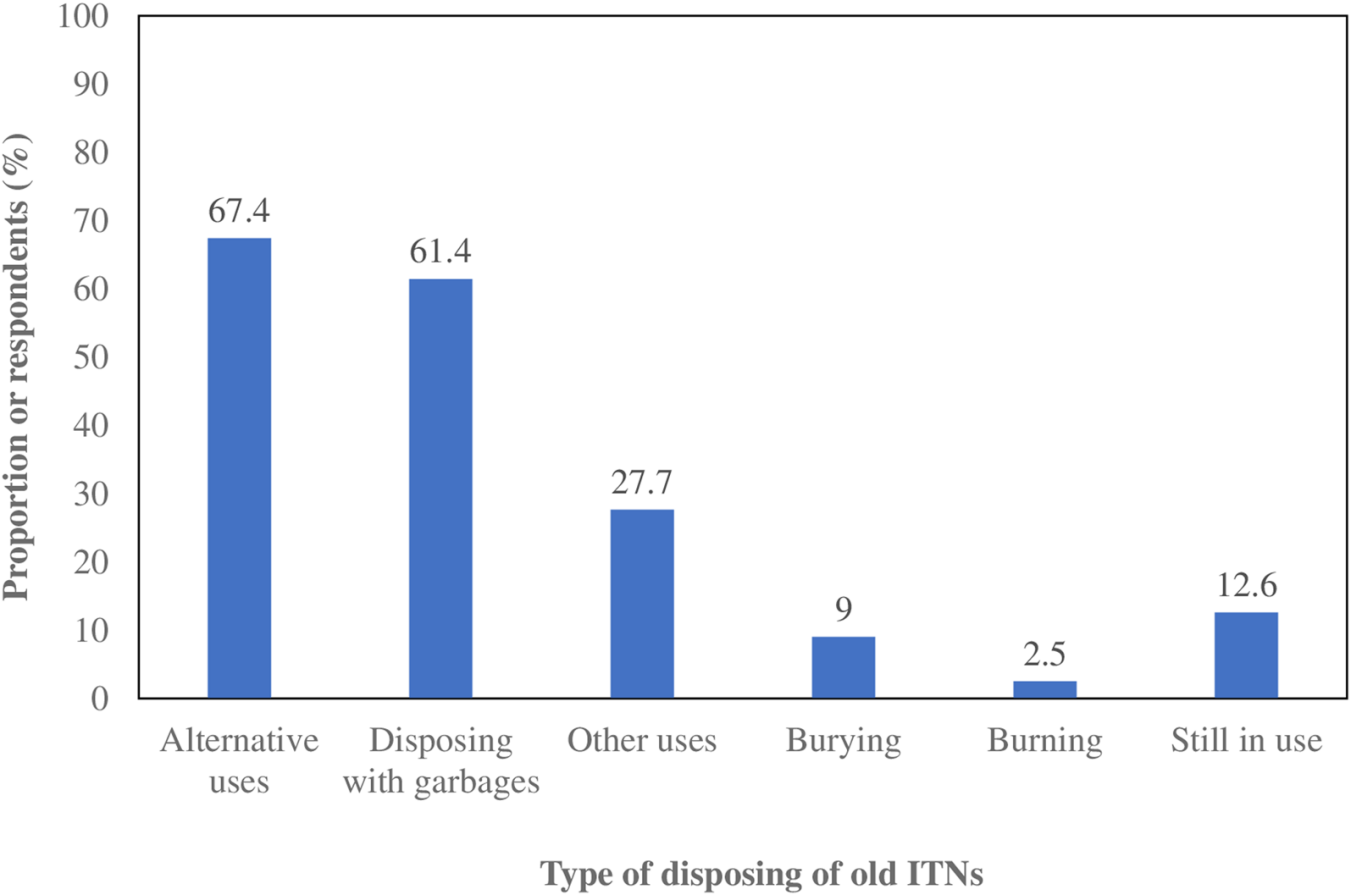
Disposal methods of old ITNs

Of the 3780 households surveyed, 87.4% (3330) reported having disposed of their ITNs when they were no longer usable due to age or wear (inactive nets). However, 12.6% (477) were still using their ITNs (active nets). The most commonly cited disposal methods included alternative uses (67.4%), disposal with other garbage (61.4%), and burying (9%). Burning was the least common method, with only 2.5% of respondents reporting it as a disposal method (Fig. 2). Responses regarding ITN disposal varied significantly by climate zone. In the Sudan zone, burning (64.8%) and burying (55.2%) were the most frequently used disposal methods. In the Sudano-Sahelian zone, other uses (39.8%), alternative uses (39.4%), and disposal with garbage (33.1%) were the predominant methods (Table 2). In the Sahelian zone, other uses (31.4%) and disposal with other garbage were the most common disposal mechanisms. Furthermore, the Table 3 presents results of univariate and multivariate analysis reported on the most common disposal method (alternative use of old ITN). Indeed, in univariate analysis, findings showed that participants with no formal education and living in rural settings were significantly 4 times (OR = 4; 95% CI 2.19–4.34) and 3.9 times (OR = 3.9; 95% CI 2.81–4.95), respectively more likely to use old insecticide-treated nets for alternative purposes as disposal method compared to those with higher education and living in urban areas (Table 3). However, according to climate areas, no significant variation was observed between participants residing in Sahelian and Sudan-Sahelian compared to Sudan area in term of alternative use of old ITN as disposal method. Moreover, after adjusting for age and wealth index, the associations were significant between no formal education and alternative use of old ITN (aOR = 3.8; 95% CI 2.13–4.32) and between living rural area and alternative use old ITN (aOR = 3.4; 95% CI 2.02–4.25]) as disposal method. However, they become less pronounced indicating a weaker effect after adjustment.

**Table 2:** Disposal methods of old ITN categorized by climate zone

Variables	Total respondents	Positive response % (*n*)	Sudan % (*n*)	Sudan-sahelian % (*n*)	Sahelian % (*n*)
Alternative uses	3780	67.4 (2548)	34.4 (878)	39.4 (1005)	26.2 (665)
Disposing with garbage	3780	61.4 (2320)	36.8 (853)	33.1 (769)	30.1 (698)
Burning	3780	9 (341)	64.8 (221)	16.7 (57)	18.5 (63)
Burying	3780	2.5 (96)	55.2 (53)	23.9 (23)	20.9 (20)
Others uses	3780	27.7 (1047)	28.7 (301)	39.8 (417)	31.4 (329)

**Table 3:** Factors influencing different uses of old ITNs

	Univariate	Multivariate
Variables Dependent vs. independent	Crude Odds Ratio [95% CI]	Adjusted Odds Ratio [95% CI)
Dependent variable “Alternative use of old ITN” as most common disposal method		
Education status		
Higher	Reference	Reference
No formal education	4*** [2.19–4.34]	3.8*** [2.13–4.32]
Primary	2.5*** [1.87–3.05]	2.1*** [1.85– 3.03]
Secondary	0.6* [0.42–0.76]	1.1* [0.69– 1.42]
Place of residence according to territorial administration		
Urban	Reference	Reference
Rural	3.9*** [2.81–4.95]	3.4** [2.02–4.25]
Climate zone		
Sudan	Reference	Reference
Sahelian	0.4* [0.23–0.62]	0.5* [0.81– 1.05]
Sudan-Sahelian	0.8** [0.63–1.29]	0.7** [0.61–1.38]
**Dependent variable “Crops and seedling fencing” as most common alternative use**		
Education status		
Higher	Reference	Reference
No formal education	5.1*** [3.84–6.1]	5.4*** [3.48–6.49]
Primary	3.9*** [2.58–4.4]	3.8*** [2.57–4.1]
Secondary	1.5** [1.01–2.48]	1.2** [0.8–1.64]
Place of residence according to territorial administration		
Urban	Reference	Reference
Rural	4.1*** [2.75–4.85]	4.9*** [3.09–5.25]
Climate zone		
Sudan	Reference	Reference
Sahelian	0.39** [0.26–0.53]	0.43*** [0.39–0.73]
Sudan-Sahelian	0.6*** [0.31–0.83]	0.62*** [0.35–0.89]

***p < 0.01, **p < 0.05, *p <0 .1

The majority of focus group discussions (FGDs) indicated that old ITNs are not systematically discarded or burned.

*“Most people sell their old ITNs to wood sellers*,* gardeners*,* or livestock farmers”* (S.A., male, aged 31, Sahelian zone).

These comments suggest that when ITNs reach the end of their useful life, they are repurposed for other uses.

The interviews also revealed that, a mosquito net is only deemed worn out when it is torn, dirty, or fails to keep mosquitoes out. Some interviewees reported discarding the nets, while others did not. In cases of unforeseen circumstances, such as the arrival of a guest or rapid wear and tear of a new net, they are kept for future use. One FGD participant explained it in the following words:

“*It’s advisable to take care of your old mosquito net because the new one might also be of poor quality*,* and the government has not yet distributed new mosquito nets.*” (R.P., woman, aged 47, Sudan zone).

### Alternative use of old ITNs

**Fig. 3:**
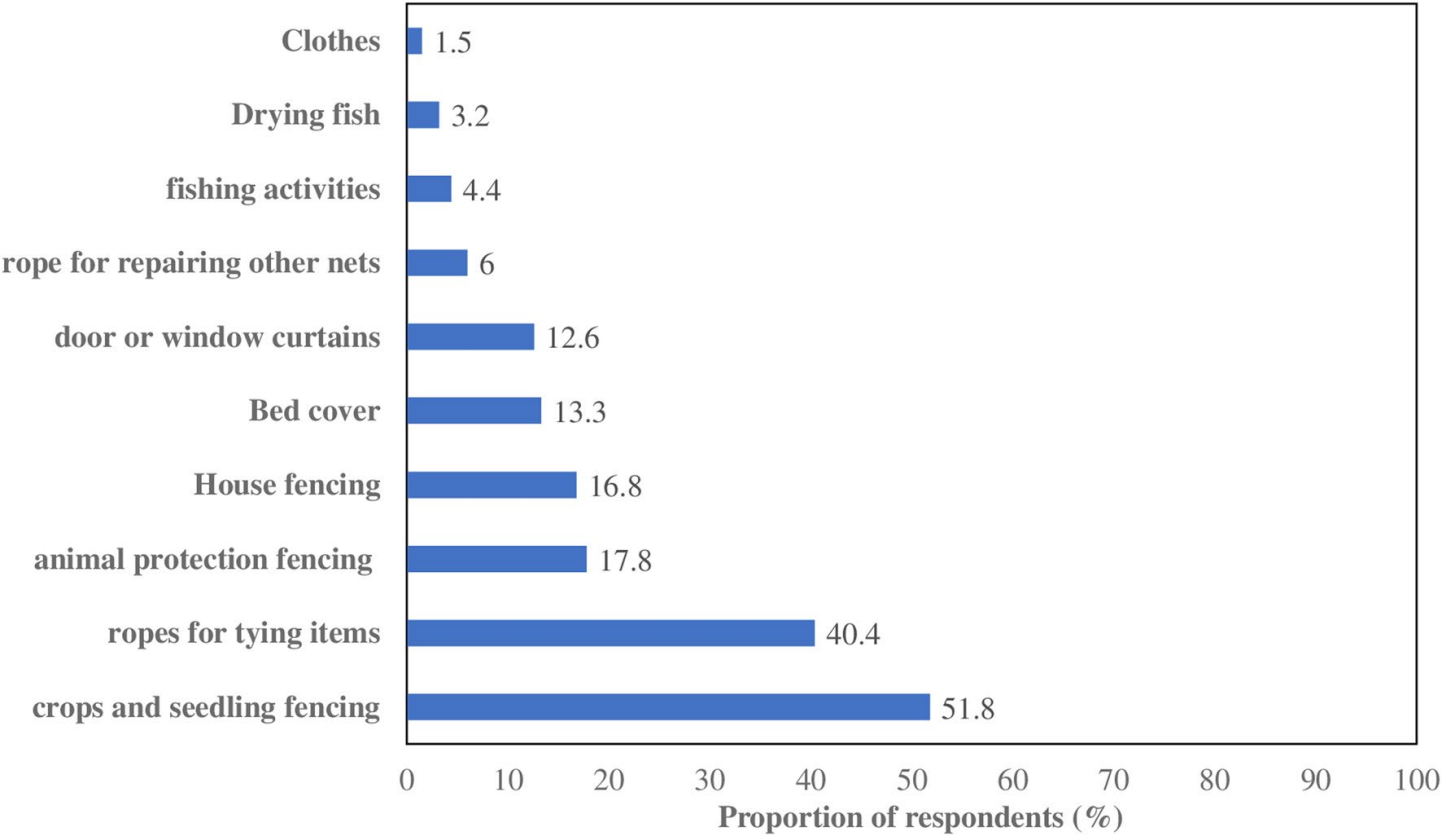
Alternative uses of old ITNs self-reported by respondents at study sites

**Fig. 4:**
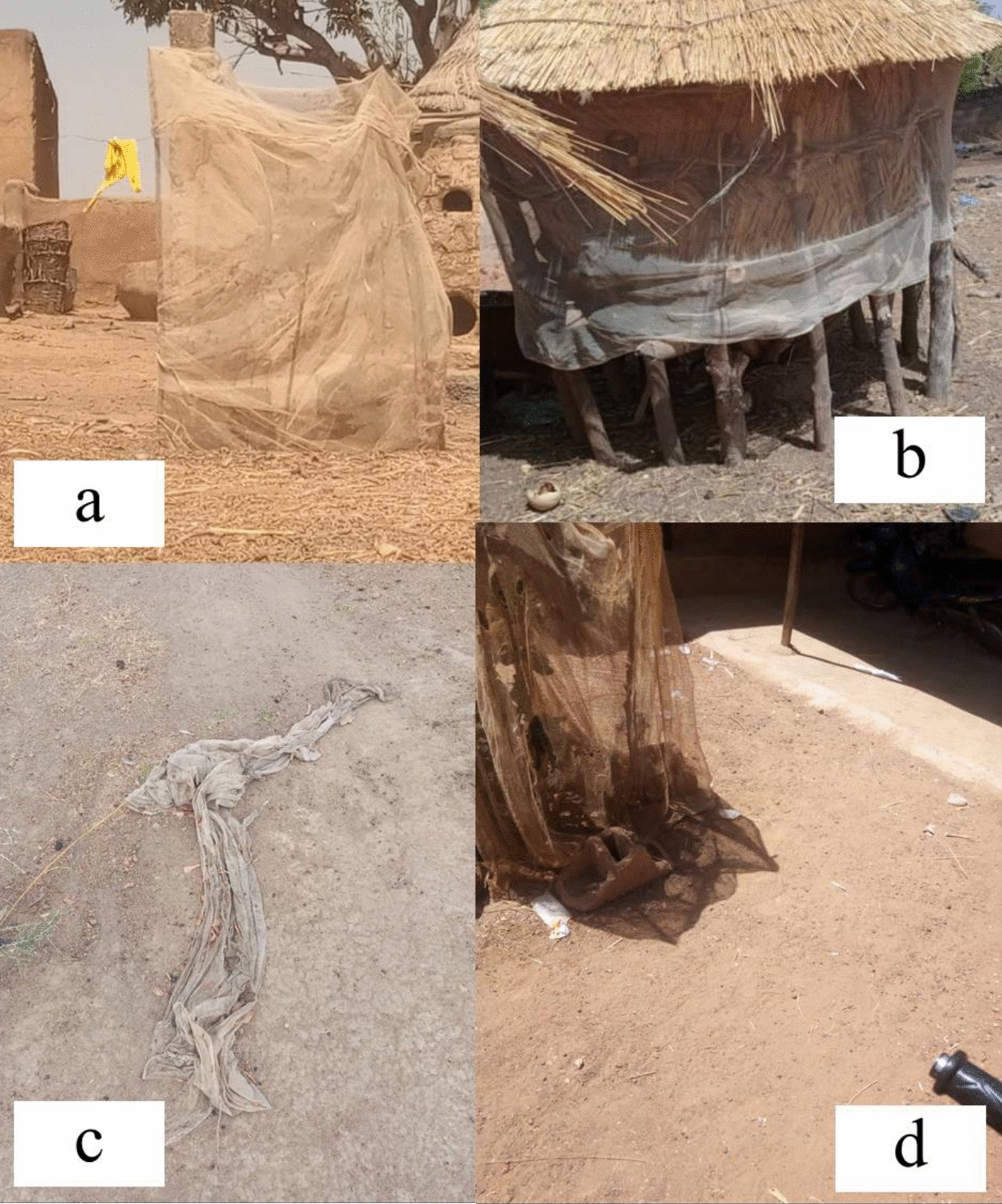
Typical examples of alternative uses for old ITNs observed by the field team at the study sites: **a** ITNs repurposed as fencing for crops and seedlings, **b** Pieces of ITN used for house fencing, **c** ITNs used as ropes to tie objects, and **d** ITNs used as door curtains

According to the survey, respondents reported several alternative uses for old ITNs that were either observed or practiced in their villages, including 51.8% (*n* = 2548) fencing for Crops and Seedlings (Fig. 3a); 40.4% (*n* = 2548) ropes for tying items (Fig. 3c): 17.8% (*n* = 2548) animal protection fencing; 16.8% (*n* = 2,548) house fencing (Fig. 3b); 13.3% (*n* = 2548) bed covers; 12.6% (*n* = 2,548) curtains for doors or windows (Fig. 3d). These findings are summarized in Fig. 4 and illustrated in Fig. (a, b, c, d). Other minimal alternative uses (7.5%; *n* = 239) reported included ropes for repairing nets, fishing activities, drying fish and sleeping clothes.

Additionally, across all climate zones, respondents reported various types of alternative uses for old ITNs, though the proportions varied. In the Sudan-Sahelian Zone, the most common alternative uses were for fencing crops and seedlings, making ropes for tying objects, and using ITNs as bed covers. However, in the Sahelian zone, common uses included fencing for animal protection, using ITNs as door or window curtains, making ropes for repairing other nets, and ITNs used for fishing, drying fish, and as clothing. In the third climate zone (Sudan zone) alternative uses were observed but at lower proportions compared to the Sudan-Sahelian and Sahelian zones (Table 4). Otherwise, the socio-demographic factors influencing the most common alternative use of old ITN (crops and seedling fencing) in all climate area were also examined in univariate and multivariate models (Table 3). Indeed, crops and seedling fencing as most common alternative use of old ITN was significantly and strongly associated with no formal education (OR = 5.1; 95% CI 3.84–6.1), primary education level (OR = 3.9; 95% CI 2.58–4.4) and resided in rural areas (OR = 4.1; 95% CI 2.75–4.85). In multivariate analysis after adjusting for age and wealth index, the increased odds ratio was significantly observed in individuals with no formal education (aOR = 5.4; 95% CI 3.48–6.49) and residing in rural area (aOR = 4.9; 95% CI 3.09–5.25) (Table 3). Indeed, Age and wealth index significantly affected the likelihood of using old ITN for crops and seedling fencing as alternative purposes after adjustment, suggesting both factors were major factors.

**Table 4:** Types of alternative uses of old ITNs self-reported by respondents categorized by climate zones

Variables	Total respondents	Positive response % (*n*)	Sudan % (*n*)	Sudan-Sahelian % (*n*)	Sahelian % (*n*)
Crops and seedling fencing	2548	51.8 (1321)	15.3 (202)	46.5 (615)	38.2 (504)
Ropes for tying items	2548	40.4 (1029)	39.8 (410)	47.3 (487)	12.9 (132)
Animal protection fencing	2548	17.8 (454)	22.2 (101)	31 (141)	46.8 (212)
House fencing	2548	16.8 (428)	29.2 (125)	31.5 (135)	39.3 (168)
Bed cover	2548	13.3 (338)	12.7 (43)	63.6 (215)	23.7 (80)
Door or windows curtains	2548	12.5 (320)	8.7 (28)	44.7 (143)	46.6 (149)
Rope for repairing other nets	2548	6 (154)	18.8 (29)	38.9 (60)	42.3 (65)
Fishing activities	2548	4.4 (113)	17.7 (20)	31.8 (36)	50.5 (57)
Drying fishes	2548	3.2 (82)	10.9 (9)	15.8 (13)	73.3 (60)
Sleeping cloches	2548	1.4 (37)	10.8 (4)	27 (10)	62.2 (23)

In addition to the alternative uses of old ITNs mentioned above in household interview and direct observation of field team (Fig. 3a, b, c and d), focus groups identified additional uses specific to each community. Notably, some of these alternative uses were related to gender roles.

For men group discussions, G.N. (male, primary school level, 30 years old, Sahelian zone) noted: *“Here*,* people tie them up to keep hay for the animals”.* Another interviewee mentioned:

“*Old ITNs are used by motorbike taxis to cover cabbages during transport*” (S.M., male, community health worker with 12 years of experience, 49 years old, Sudan zone). These comments indicated that men used old ITNs for economic purposes.

In the women’s category, the interviews revealed that old ITNs are used to store kitchen utensils and other objects in the house, or to cover vegetables and food during transport. Two women explained in the following words during FGDs:

“*Women repurpose old mosquito nets to tidy up their homes by using them to tie up their own items such as crockery and clothes*” (S.B., female, no formal education, 49 years old, Sudan zone).

“*They also use the nets to cover food and vegetables when transporting them to sell at the market*” (O.S., female, secondary education, 18 years old, Sudano-Sahelian zone).

In addition, two FGD participants indicated:

*“People in our community use these old ITNs to cover their granaries*” (S.O., female, community health worker with 7 years of experience, 52 years old, Sudan zone).

“*We use these old nets to wash our dishes and ourselves. Sometimes*,* during the rainy season*,* the children use them to catch small fish and bring them back for us to cook*” (S.D., woman, 38 years old, Sahelian zone).

In addition to the socio-economic uses of old ITN described by men and women above, another uncommon type of alternative use of old ITNs was reported in Sudan area.

K.I. (male, 46 years old, secondary education, Sudan zone) said, “*We use these old mosquito nets to cover corpses*”.

These comments highlight the diverse ways in which old ITNs are alternatively used by communities in Burkina Faso. Respondents provided several reasons for alternative uses of old ITNs in their community. The primary reason is the poor condition of the nets, particularly those made of hard fabric of fibers. Indeed, interviewees expressed concerns about perceived discrimination in the choice of fabric for mosquito nets. Some people also reported using even new nets for alternative purposes if they find them unsuitable for their intended use. One respondent (S.D., female, aged 63, from the Sudano-Sahelian zone) remarked, “*You choose the good ones to give to your families and you give us the bad ones*.” This feeling may help explain why the nets are often repurposed for other uses.

## Discussion

The findings revealed that the predominant disposal practices for ITNs include repurposing them for alternative uses and discarding them with regular garbage. Similar alternative uses of ITNs have been reported in other parts of Sub-Saharan Africa [23–28]. An unconventional alternative use reported in the study involved covering the bodies of deceased persons. Consistent with other research on the reasons for alternative uses, this study found that the main reason for repurposing old ITNs was their worn condition. Participants also mentioned that the nets were unsuitable for protection against mosquitoes due to their large mesh (holes) and perceived poor quality. Preferences based on brand, mesh size, and net color have been shown to influence total ITN coverage in many other settings in sub-Saharan Africa [26, 29, 30]. Programmes aimed at increasing ITN coverage and proper usage must consider local community preferences.

The widespread alternative uses of insecticide-treated nets pose risks to living organisms. For instance, this study found that community members used ITNs as fencing to protect crops and animals, as ropes to tie things up, as bed covers, and even as bath sponges. If these nets still contain insecticide residues, they pose a risk not only to human health but also to the environment, especially when old nets are thrown away with household garbage. Indeed, residual insecticides from discarded nets can hinder malaria elimination efforts by contributing to mosquito resistance to insecticides and environmental pollution [7]. As observed in other studies, ITNs are frequently repurposed for uses other than mosquito control. Research conducted in various countries has documented a wide range of alternative uses, including protecting cabbage crops on small farms, shrimp fishing, safeguarding seedlings and vegetable crops, braiding twine to tie livestock, using the nets as washing sponges, covering meat in butcheries, filtering water, and as window curtains, or for protecting crops and chickens [26, 31–34]. These practices, already in place in many countries, could serve as a foundation for developing guidelines for the disposal and alternative use of old ITN, tailored to different contexts. Collaborative efforts between government authorities and community members in designing and implementing projects and programmes could lead to smoother operations and the development of sustainable, long-term solutions to a variety of challenges.

Many studies investigating ITN disposal and alternative use have primarily focused on specific regions, particularly in East Africa, with limited research in West Africa, including Burkina Faso. For instance, studies in Tanzania and Kenya have dominated the literature on ITN usage, disposal, and repurposing [19, 35]. However, these findings may not fully translate to Burkina Faso, which has diverse climate zones ranging from Sahelian to Sudan-Sahelian, potentially influencing ITN use and disposal behaviors differently. A study by Feng et al. [36]. identified improper disposal methods in East Africa but did not examine the disposal practices in West Africa. Furthermore, socio-demographic factors such as education level, wealth status, and rural-urban residency significantly influence health behaviors, including ITN use and disposal practices. However, few studies have systematically analysed how these factors affect ITN disposal behaviours. Research tends to overlook the roles of socio-economic disparities, and education, as well as the different practices in urban versus rural settings [37]. For example, Baume et al. [32] analysed ITN use patterns but did not fully explore the implications of socio-demographic characteristics on disposal. Similarly, Koenker et al. [38] found that education and wealth influenced ITN retention and misuse but stopped short of exploring how these factors affect proper disposal and alternative uses. This creates a knowledge gap, particularly in West African settings, where socio-demographic factors could vary significantly across different climate zones. This current study has overcome this gap in the literature by providing significant findings through distribution of disposal method and improper alternative use of old ITN according to climate zone (Sudan, Sudan-Sahelian and Sahelian). In addition, this study has also examined the socio-demographic factors which could be influence of different use of old ITN in Burkina Faso. The key findings showed that the education status and place of residence of participants were strongly correlated to most common disposal method (alternative use) and type of alternative use of old ITN (crops and seedling fencing).

The study suggests that addressing these issues requires community-level interventions. These interventions should include education on the proper disposal of used nets and the promotion of alternative income-generating activities to discourage the misuse of nets. However, health promoters should provide guidance on alternative uses, such as encouraging the use of nets in ways that complement malaria control efforts, like using them as window screens or to cover water wells to prevent mosquitoes from emerging. Additionally, efforts should be made to involve communities in developing viable and realistic methods of reusing old nets. This could be achieved without compromising the overall objectives of malaria control initiatives. For example, old nets could serve as an alternative source of income by promoting the collection, sorting, and manufacturing of ropes, which could then be sold to generate additional household income.

### Key findings and recommendations

#### Summary of key findings

A significant number of old ITNs are disposed of with household garbage and although less common, burning is still reported as a disposal method. Improper disposal and burning contribute to environmental pollution, potentially harming ecosystems and public health. On the other hand, the majority of old ITNs are repurposed for various functions such as fencing, tying objects, and covering food. However, alternative uses, such as using ITNs as ropes or for food coverage, may expose individuals to residual insecticides, posing risks to human health and the environment. The physical condition of the net, particularly its level of wear and tear and type of net fibers as well, significantly influences its repurposing, with severely damaged nets being more likely to be reused. Furthermore, the absence of clear disposal guidelines has led to inconsistent and often informal disposal methods, contributing to both health risks and environmental pollution.

#### Recommendations for managing old ITNs

*Development of clear guidelines at national level for the disposal and alternative use of old ITNs*.

Establish and widely distribute clear guidelines for the disposal and repurposing of old ITNs. These guidelines should outline safe disposal methods and recommend alternatives that ensure both health and environmental safety. Additionally, implement educational programmes to teach communities about proper disposal practices and the risks associated with improper disposal.

#### Promote safe alternative uses of old ITNs

Encourage the repurposing of old ITNs in ways that support malaria control goals, such as using them as window screens or to cover water sources. In addition, support initiatives that convert old nets into products like ropes or covers that can be sold, providing economic benefits while ensuring proper reuse.

#### Community involvement in old ITN disposal and alternative uses

Engage community members actively in creating and implementing disposal and repurposing strategies. Their insights can help develop more practical and widely accepted solutions. Moreover, collaborate with local organizations and authorities to establish sustainable programmes that address both health and socio-economic needs.

#### Research on impact of old ITN disposal and alternative uses

Continuously research the effects of different disposal methods on health and the environment and regularly update and adjust practices based on new evidence and community feedback.

## Conclusion

The study on the disposal and alternative use of old insecticide-treated nets (ITNs) in Burkina Faso highlights a complex interplay between practical needs, community practices, and health considerations. The findings emphasize the need for comprehensive strategies to manage the disposal and repurposing of old ITNs in Burkina Faso. By addressing the gaps in guidelines, promoting safe and beneficial reuse practices, and involving communities in the process, it is possible to mitigate health risks and enhance the effectiveness of malaria control programmes.

## Data Availability

The datasets used and/or analyzed during the current study are included in the manuscript.

## Declarations

### Significance

****p* < 0.01, ***p* < 0.05, **p* < 0 0.1.
